# The VIP trial: a randomised controlled trial of the clinical effectiveness of a Victim Improvement Package (VIP) for the reduction of continued symptoms of depression or anxiety in older victims of community crime in an English city

**DOI:** 10.1136/bmjopen-2024-095184

**Published:** 2025-07-25

**Authors:** Marc Serfaty, Jessica Satchell, Teresa Lee, Gloria Laycock, Chris Brewin, Marta Buszewicz, Gerard Leavey, Vari M Drennan, Victoria Vickerstaff, Jonathan Cooke, Anthony Kessel

**Affiliations:** 1 University College London, London, UK; 2 Priory Hospital North London, London, UK; 3 Department of Psychology, University of Ulster, Derry, UK; 4 Kingston University, London, UK; 5 PPI member, London, UK; 6 LSHTM, London, UK

**Keywords:** MENTAL HEALTH, PSYCHIATRY, PUBLIC HEALTH, Quality of Life, Randomized Controlled Trial, Crime

## Abstract

**Background:**

Older crime victims may be particularly vulnerable to psychological distress.

**Objectives:**

To compare the clinical effectiveness of a Victim Improvement Package (VIP) to treatment as usual (TAU) for reducing continued crime-associated distress.

**Design:**

A three-step parallel-group single-blind randomised controlled trial.

**Setting:**

Police-reported victims of community crime aged 65 and over were recruited from 12 local authority areas in a major urban city in England, UK.

**Participants:**

Selection criteria—inclusion: victims of community crime aged 65 years or more, with significant Generalised Anxiety Disorder (GAD-2) and Patient Health Questionnaire (PHQ-2) distress associated with the crime. Exclusion: type of crime, diagnosis, receipt of cognitive–behavioural therapy (CBT) in the last 6 months; an inability to participate in CBT; cognitive impairment. Participants were typical of our local authority population; 71% were female, 69% white, with the majority of crimes associated with burglary (35%) and theft (26%). 67% (88/131) of the randomised participants were included in the primary analysis.

**Interventions:**

TAU was compared with TAU plus up to 10 sessions of a cognitively-behaviourally informed VIP, delivered by a mental health charity over 12 weeks.

**Primary and secondary outcome measures:**

Timings are in relation to the crime; baseline (3 months), post-VIP intervention (6 months) and follow-up (9 months). The primary outcome was a composite of the Beck Anxiety and Beck Depression Inventories. The primary endpoint was 6 months.

**Results:**

24% (4255/17 611) of reported crime victims were screened, 35% (1505/4255) were distressed. Of 60% (877/1505) rescreened at 3 months, 49% (427/877) remained distressed. Out of our target of 226, 131 participants were randomised; 65 to VIP and 66 to TAU alone. 68% (89/131) completed the primary outcome (post-intervention). The VIP showed no overall benefit; mean VIP −0.41 (SD 0.89) vs mean TAU −0.19 (SD 1.11); standardised mean difference −0.039; 95% CI (−0.39, 0.31), although stratified analyses suggested an effect in burglary victims (n=27, standardised mean difference −0.61; 95% CI (−1.22, –0.002), p=0.049).

**Conclusions:**

Community crime had long-lasting impacts. The police are ideally placed to screen for distress, present in 35% of victims, but only 58% of participants were recruited and the cognitive–behavioural therapy was not delivered competently. Further research on victim care and improving the delivery and quality of therapy is required.

**Trial registration number:**

All procedures were approved by the University College London (UCL) Research Ethics Committee on 17 March 2016 (6960/001). International Standard Randomised Controlled Trial Number is ISRCTN16929670: https://doi.org/10.1186/ISRCTN16929670.

**STRENGTHS AND LIMITATIONS OF THIS STUDY**

- Largest study of victims of community crime, although police reported crime only.
- Randomised controlled trial design.
- Standardised selection criteria and outcome measures were used.
- The treatment was manualised.
- Adherence and quality of therapy assessed.

## Background

 The world’s population is ageing^1^ with depression and anxiety common among older people.^2^ Frailty, disrupted personal ties and loneliness make them vulnerable to the psychological impact of crime.^3^ Recent evidence^4^ suggests psychological distress is prevalent among older victims, with anxiety, depression and trauma symptoms commonly reported. Our feasibility study found 27% of older victims had continued symptoms at 3 months.^5^ The social impact and health implications of crime may also be significant, with being a victim of crime in late-life being associated with a twofold increase in risk of dying or care-home placement.^6 7^

Cognitive–behavioural therapy (CBT) is effective for depressive symptoms (with or without anxiety) in older people^8^ and although psychological therapies are preferred to medication,^9^ they are rarely offered.^10 11^ Only four psychological interventions for distress in older victims have been published, and all were feasibility studies^4^; two nursing schemes,^12 13^ a psychoeducation video^14^ and our pilot randomised controlled trial (RCT), the Helping Aged Victims of Crime (HAVoC) study,^5^ which developed and piloted a manualised CBT informed Victim Improvement Package (VIP).^15^

Further to the HAVoC study, we collaborated with the police force and a national mental health charity in a large metropolitan area to develop the VIP trial^16^; an RCT into the clinical and cost effectiveness of this intervention for treating anxiety and depressive symptoms in older victims of crime.

During the trial, screening and thus recruitment were challenging, despite practical adaptations. Several factors were external to our control including re-deployment of police staff from community policing after major events; terrorism and climate change activism, the COVID-19 pandemic and decreased confidence in the police made the participant recruitment increasingly untenable, obliging the premature end of the trial. We report on the key findings from the trial which, although unable to provide evidence on effectiveness, are nevertheless important.

### Objectives

To conduct a randomised controlled trial of the clinical and cost effectiveness of a VIP for the reduction of chronic symptoms of depression and/or anxiety in older victims of community crime.

## Methods

**Figure 1:**
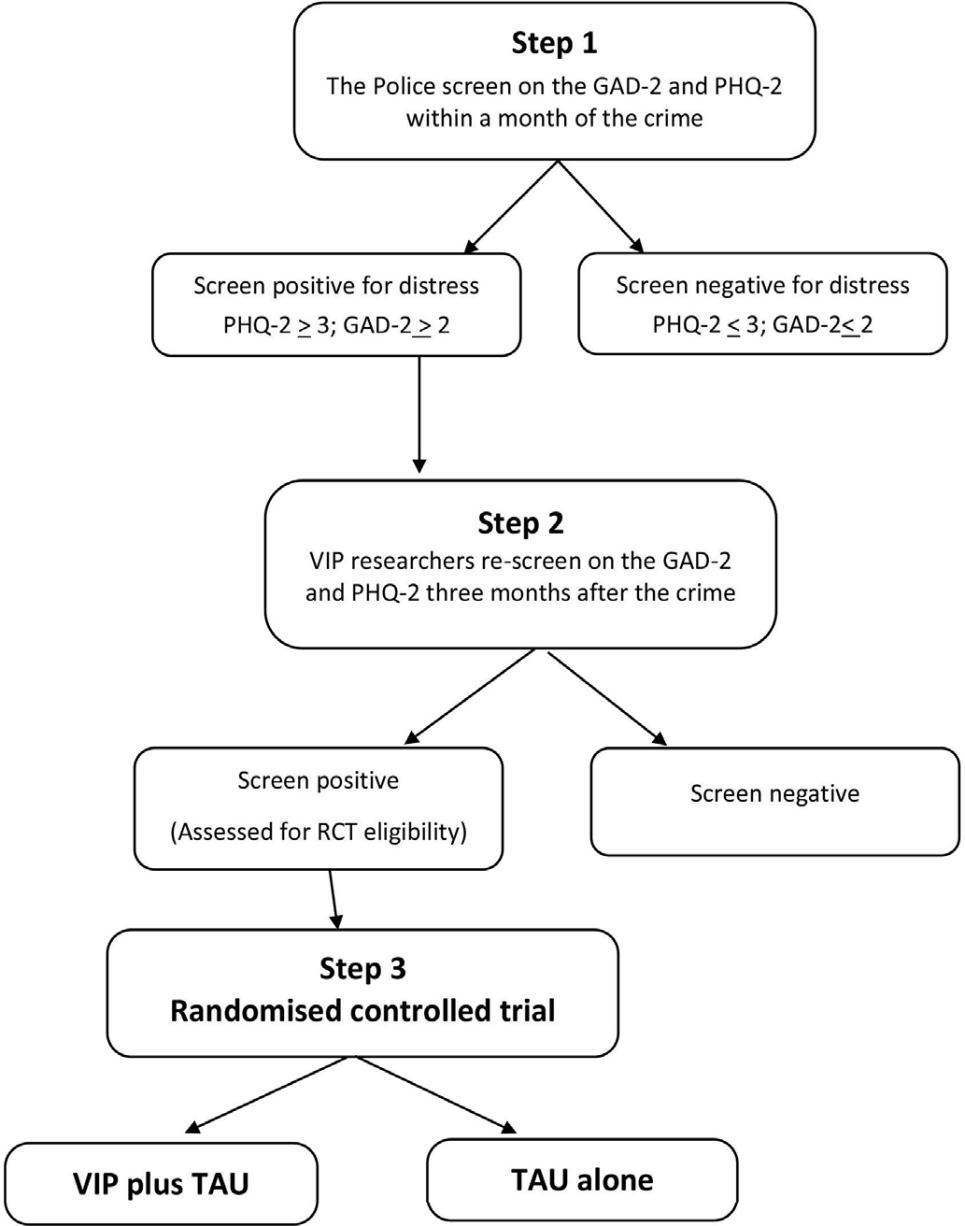
Flowchart of the VIP trial design. GAD-2, Generalised Anxiety Disorder; PHQ-2, Patient Health Questionnaire; RCT, randomised controlled trial; TAU, treatment as usual; VIP, Victim Improvement Package.

A summary of the main design and amendments of the published protocol^16^ is given below (figure 1).

### Design

Parallel group, single-blind, individually randomised controlled trial comparing treatment as usual (TAU) with TAU plus up to 10 sessions of a VIP.

The trial consisted of three steps outlined in figure 1.

The VIP Trial was registered with the University Data Protection Office on 26 February 2016. The authors assert that all procedures contributing to this work comply with the ethical standards of the relevant national and institutional committees on human experimentation and with the Helsinki Declaration of 1975, as revised in 2008. All procedures involving human participants were approved by the University College London (UCL) Research Ethics Committee on 17 March 2016 (6960/001). Signed informed consent was obtained for all participants.^2 3^ International Standard Randomised Controlled Trial Number is ISRCTN16929670: https://doi.org/10.1186/ISRCTN16929670.

### Public participant involvement

Two older adults with experience of CBT and the impact of crime provided feedback on the project materials, ethics and write-up. They attended regular trial management and steering group meetings.

### Setting

#### Location and timing of study

Recruitment commenced May 2017 and was extended on three occasions to end September 2022. The first participant was randomised on 20 November 2017 and the final participant, final follow-up was on 16 February 2023. The study was conducted in a large urban area, including inner and outer city areas expanded to 12 local authority areas selected for the range of demographic characteristics of their populations.

#### Population

Police-reported victims of community crime aged 65 and over, with continued symptoms of anxiety and/or depression were identified using the procedures described below. The WHO^2^ defines community violence as that perpetrated by strangers or acquaintances and this definition was extended to all crime types. Crimes committed within relationships where there is an expectation of trust^17^ (eg, domestic violence, carer abuse) were excluded. All types of sexual violence, whether known to the victim or not, were also excluded, given a more specialist intervention may be needed.^5^

### Eligibility and screening

We used the Patient Health Questionnaire (PHQ-2)^18^ and Generalised Anxiety Disorder (GAD-2)^19^ questionnaire for screening. Each question scores from 0 to 3. The total PHQ-2 is the sum of the scores (range 0–6). The GAD-2 also consists of two questions and is scored in the same way. Victims were defined as screen positive for distress if they had significant depressive (>3 for the PHQ-2) and/or anxiety symptoms (>2 for the GAD-2). As the trial progressed, three different screening approaches (intermittently between June 2017 and June 2023) were used, adapting our methods to changing circumstances.^20^

### Step 1 – police screening

Screen methodology 1 (SM1) (June 2017 to August 2019) used Safer Neighbourhood Teams (SNTs). SNTs typically consist of one dedicated Sergeant, a Police Constable and Police Community Support Officers, working across several local authority areas. They aimed to visit as many older victims of community crime as possible, screen them for distress and, if significant for distress, gain consent to refer them to the university research team who undertook step 2 outlined below.

In methodology 2 (SM2) (May 2021 to June 2022), two police staff identified and screened over the telephone and notified SNTs to visit screen-positive victims at home. The same procedures as in SM1 were then undertaken.

In methodology 3 (SM3) (17 March to 30 June 2023), two police administrators made telephone contact with the victim and, with their agreement, passed their details to a specialist university researcher integrated within the police service who screened participants for distress and then provided them with information about the trial and direct SNTs to undertake a home visit using the same procedures as in SM1.

All distressed victims were signposted to their general practitioner (GP). Because of pressures on staff resourcing and possibly poorer public confidence in the police, recruitment became too limited to proceed with SM3 and screening (and thus recruitment) had to be terminated. Therefore, only those from screening SM1 and SM2 proceeded to step 2 and are included in this paper.

### Step 2 rescreening (3 months post crime)

Those screening positive at step 1 were rescreened for significant distress by a university researcher as part of step 2 using the PHQ-2 and GAD-2. This was initially through home visits then by telephone during the COVID-19 pandemic.

### Step 3 entry into the VIP trial (3 months post crime)

#### Participants. Selection criteria

Inclusion: victims of community crime aged 65 years or more, with scores above cut-offs on the GAD-2 and PHQ-2 and who associated their distress with the crime.

Exclusion: victims of sexual violence or domestic violence; a self-reported diagnosis of schizophrenia or bipolar disorder; a Mini International Neuropsychiatric Interview (MINI)^21^ diagnosis of alcohol dependency; receipt of CBT in the last 6 months; an inability to participate in CBT because of language difficulties; significant cognitive impairment, indicated by a 6-item Cognitive Impairment Test score of 10 or more.^22^

### Randomisation

We used a web-based system (Sealed Envelope- www.sealedenvelope.com) to randomise participants to either TAU or TAU plus the VIP. Randomisation was undertaken using permuted blocks of variable size, stratifying participants according to the presence of symptoms of depression with/without anxiety or anxiety alone according to PHQ-2/GAD-2 scores. Other than an independent trial administrator conducting the randomisation, all research staff were blind to the group allocation; participants could not be masked to their treatment allocation.

### The interventions

#### Control - Treatment as Usual (TAU)

TAU could consist of informal support provided by networks of friends, relatives, voluntary agencies or through self-referral to the mental health charities. Although victims could self-refer to their GP or be referred to more formal services such as Improving Access to Psychological Therapies (IAPT), traumatic stress clinics or private psychological support, our experience was that this was rare^23^ and access to IAPT services was often associated with delays of several weeks.^24^

#### Intervention -The Victim Improvement Package (VIP)

Up to 10 manualised individual sessions of modified CBT were offered over 3 months, by a community-based mental health charity, initially face-to-face in a community setting, then remotely during the COVID-19 pandemic and following this, a choice of delivery. “The VIP intervention consists of Up to 10 manualised individual sessions were delivered over 3 months by mental health charity therapists. These are: Session 1: Crime narrative, underlying beliefs, behaviour; Session 2: Psychoeducation about crime and CBT; sessions 3–8: mood diaries, guided discovery, behavioural experiments; sessions 9–10: relapse prevention. These are summarised further in^16^ full in the VIP manual, available from the Chief Investigator ^15^.”

Therapist characteristics: we aimed to use therapists accredited by the British Association of Behavioural and Cognitive Psychotherapists (BABCP) or with at least 2 years’ experience in CBT.

Training: 49 therapists attended a 1 day training session to help adapt their skills in older victims of crime. They were familiarised with the training manual and practised case examples. Six therapists took up the offer of a 6 months’ booster session.

Supervision of therapists: this was offered every 2 weeks by the Chief Investigator, MS.

Measures of fidelity: therapists were asked to upload the following onto a secure database (DataSafeHaven): (a) Digital recordings of each session with (b) a Therapy Component Checklist (TCC) (see online supplemental materials) to rate therapy quality using the Cognitive Therapy Scale-Revised^25^ and adherence using the TCC.^26^ A random sample of 15% of uploads was rated.

### Measures

#### Demographic characteristics

Sociodemographic data were collected by the police using a trial pro-forma. This included gender, age, ethnicity, crime reported, social information and perception of impact of crime (table 1).

**Table 1:** Summary of timing of measures

	Baseline (3 months)	Post-intervention (6 months)	Follow-up (9 months)
BDI-II	**✓**	**✓**	**✓**
BAI	**✓**	**✓**	**✓**
MINI (caseness)	Yes/no	Yes/no	
EQ-5D	**✓**	**✓**	**✓**
CSRI (including psychological therapies and prescribed psychotropic medication)	**✓**	**✓**	**✓**
Satisfaction with VIP (VIP group only)		**✓**	
Expectation of therapy	**✓**		
Blindness to group allocation by research assistant		**✓**	**✓**
Attrition and reason		**✓**	**✓**
Fidelity: adherence and CTS-R		**✓**	

BAI, Beck Anxiety Inventory; BDI-II, Beck Depression Inventory-II; CSRI, Client Service Receipt Inventory; CTS-R, Cognitive Therapy Scale-Revised; EQ-5D, EuroQol-5D; MINI, Mini International Neuropsychiatric Interview; VIP, Victim Improvement Package.

### Primary and secondary outcome measures

The primary outcome was a composite of the Beck Depression Inventory-II (BDI-II)^27^ and Beck Anxiety Inventory (BAI).^28^ A composite measure was used as the impact of crime affects individuals in different ways, presenting with depressive and/or anxiety symptoms. Both scores were combined using the standardised BDI-II scores for participants with depression (with/without anxiety) and standardised BAI scores for participants with anxiety only, regardless of treatment allocation. Secondary outcomes were the separate scores on the BDI-II and BAI, MINI diagnoses. We collected health economics data using the EuroQol^29^ and a modified Client Service Receipt Inventory (CSRI).^30^ These findings will be published separately.

### Other measures, including measures of bias

(i) Measures of attrition and engagement with therapy: change of residence, psychiatric illness, non-attendance rates and reason for not attending. (ii) Assessment of ‘blindness’ by the rater. (iii) Changes in prescribed psychotropic medication. (iv) Other psychological treatments received. (v) Measures of fidelity to treatment. (vi) Measures of satisfaction with therapy rated on a 5-point scale. (vii) Serious adverse events (SAEs).

### Timing of outcomes

The timings of measures are presented (table 1) and all refer to time from the original crime. Baseline was at 3 months, primary endpoint was post-intervention (6 months) and the secondary endpoint at follow-up (9 months).

### Statistics

Power: To detect a (‘true’) average difference of at least 0.5 on the standardised joint scale with 90% power and a 5% significance level with a 1:1 randomised ratio, a total sample size (N) of 168 was required. This was inflated to 226 when accounting for therapist cluster effects, assuming a cluster size of 8, intra-class correlation coefficient of 0.02 and inflation size of 15%.^16^

Analysis: The primary outcome was analysed on an intention-to-treat basis using a multilevel mixed effects model allowing for facilitator clustering in the intervention arm, and baseline BDI/BAI values and study site were included as fixed effects. Each control arm individual was treated as a separate cluster. Mixed logistic regression models were used to analyse binary outcomes, allowing for therapist clustering and with the same fixed effects. The main analysis was complete case analysis. However, if participants had <20% of the items missing on a questionnaire, responses were imputed by substituting the means of the completed items.

In sensitivity analyses, the primary outcome model was refitted, adjusting for baseline predictors of missing data identified by logistic regression models. Analyses using stratification (primary diagnosis, treatment preference, deprivation and crime groups), worse and best-case scenarios, and multiple imputation (using predictive mean matching with k=20) were also performed. Stata v18.0 was used for all analyses. Health economics results will be reported separately.

## Results

**Figure 2:**
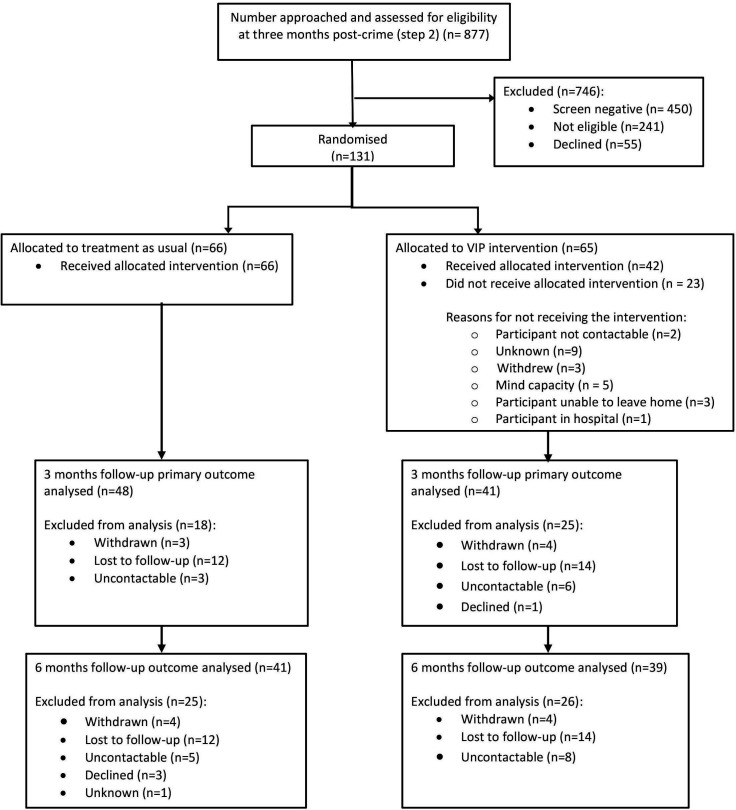
VIP CONSORT diagram. CONSORT, Consolidated Standards of Reporting Trials; VIP, Victim Improvement Package.

We screened 24% (4255/17 611) of reported community crimes in older victims and 35% (1505/4255) were significantly distressed. Of those distressed, we rescreened 60% (877/1505) at 3 months post crime and 49% (427/877) remained distressed and 44% (186/427) agreed to be considered for the VIP trial. 131 people were randomised, 66 in the TAU arm and 65 in the TAU plus VIP, as shown in the Consolidated Standards of Reporting Trials diagram (figure 2). 67% (88/131) of the randomised participants were included in the primary analysis.

Other than previous anxiety or depressive symptoms, the demographic and other characteristics of randomised participants appeared well balanced and typical of our area (table 2); 67.2% were female and 31.3% were non-white. Reported prescribed psychotropic medication was balanced between the two arms. 14% (18/131) were taking an antidepressant, 3% (4/131) an anxiolytic/hypnotic and 2% (3/131) a neuroleptic. Antidepressant use in those receiving the VIP intervention changed from 12% (8/65) to 8% (5/55) but remained unchanged in the TAU arm at 15% (10/66). For those on an antidepressant, the mean equivalent dose of clomipramine at baseline was 149.2 mg (SD 42.5) for the VIP and 112.0 mg (SD 73.4) for TAU.

**Table 2:** Demographic and other patient characteristics of randomised participants (n=131)

Variable	VIP (n=65)	TAU (n=66)	Total (n=131)
	Mean (SD) or n (%)	Mean (SD) or n (%)	Mean (SD) or n (%)
Age (years)	72.1 (5.9)	72.1 (11.2)	72.1 (8.9)
Gender			
Female	46 (70.8%)	42 (63.6%)	88 (67.2%)
Male	19 (29.2%)	24 (36.6%)	43 (32.8%)
Total	65 (100%)	66 (100%)	131 (100%)
Ethnicity			
White	45 (69.2%)	45 (68.2%)	90 (68.7%)
Black	5 (7.7%)	4 (6.1%)	9 (6.9%)
Asian	8 (12.3%)	10 (15.2%)	18 (13.7%)
Other	7 (10.8%)	7 (10.6%)	14 (10.7%)
Total	65 (100%)	66 (100%)	131 (100%)
Victim vulnerability			
Recorded vulnerability	7 (10.8%)	13 (19.7%)	20 (15.3%)
No recorded vulnerability	58 (89.2%)	53 (80.3%)	111 (84.7%)
Total	65 (100%)	66 (100%)	131 (100%)
Marital status			
Single	12 (18.5%)	11 (16.7%)	23 (17.6%)
Married/cohabitating	29 (44.6%)	23 (34.8%)	52 (39.7%)
Widow/widower	10 (15.4%)	19 (23.8%)	29 (22.1%)
Divorced/separated	13 (20.0%)	11 (16.7%)	24 (18.3%)
Other	1 (1.5%)	2 (3.0%)	3 (2.3%)
Total	65 (100%)	66 (100%)	131 (100%)
Education			
Primary	3 (4.9%)	3 (5.0%)	6 (5.0%)
Secondary	34 (55.7%)	27 (45.0%)	61 (50.4%)
Higher	24 (39.3%)	30 (50.0%)	54 (44.6%)
Total	61 (100%)	60 (100%)	121 (100%)
Living arrangement			
Rented	27 (41.5%)	29 (46.0%)	56 (42.7%)
Owner/occupier	36 (55.4%)	35 (53.0%)	71 (54.2%)
Other	2 (3.1%)	2 (3.0%)	4 (3.1%)
Total	65 (100%)	66 (100%)	131 (100%)
Crime group			
Assault	5 (7.8%)	6 (9.1%)	11 (8.5%)
Burglary	18 (28.1%)	23 (34.9%)	41 (31.5%)
Criminal damage	8 (12.5%)	13 (19.7%)	21 (16.2%)
Fraud	2 (3.1%)	1 (1.5%)	3 (2.3%)
Harassment	4 (6.3%)	3 (4.6%)	7 (5.4%)
Robbery	2 (3.1%)	3 (4.6%)	5 (3.9%)
Theft	25 (39.1%)	17 (25.8%)	42 (32.3%)
Total	64 (100%)	66 (100%)	130 (100%)
Anyone arrested?			
Yes	7 (10.8%)	3 (4.6%)	10 (7.7%)
No	58 (89.2%)	62 (95.4%)	120 (92.3%)
Total	65 (100%)	65 (100%)	130 (100%)
Affected daily life			
Yes	56 (87.5%)	62 (95.4%)	118 (91.5%)
No	8 (12.5%)	3 (4.6%)	11 (8.5%)
Total	64 (100%)	65 (100%)	129 (100%)
Previously suffered from depression or anxiety?			
Yes	29 (46.8%)	40 (61.5%)	69 (54.3%)
No	33 (53.2%)	25 (38.5%)	58 (45.7%)
Total	62 (100%)	65 (100%)	127 (100%)
Social contact	3.6 (3.5) (n’*=46)	6.6 (13.9) (n’*=60)	5.2 (10.4) (n’*=116)
Sense of safety before crime			
Very safe	13 (20.0%)	19 (28.8%)	32 (24.4%)
Safe	38 (58.5%)	28 (42.4%)	66 (50.4%)
Neither safe nor unsafe	7 (10.8%)	8 (12.1%)	15 (11.5%)
Unsafe	6 (9.2%)	10 (15.2%)	16 (12.2%)
Very unsafe	1 (1.5%)	1 (1.5%)	2 (1.5%)
Total	65 (100%)	66 (100%)	131 (100%)
Sense of safety after crime			
Very safe	0 (0.0)	0 (0.0%)	0 (0.0%)
Safe	9 (13.9%)	4 (6.1%)	13 (9.9%)
Neither safe nor unsafe	7 (10.8%)	11 (16.7%)	18 (13.7%)
Unsafe	31 (47.7%)	33 (50.0%)	64 (48.9%)
Very unsafe	18 (27.7%)	18 (27.3%)	36 (27.5%)
Total	65 (100%)	66 (100%)	131 (100%)
Severity of anxiety symptom (GAD-2)	6.4 (1.7)	6.6 (1.6)	6.5 (1.6)
Severity of depression symptoms (PHQ-2)	5.4 (1.9)	5.7 (1.9)	5.5 (1.9)

* n’ is the number of observations included in the summary for continuous variables (if different from n).

GAD-2, Generalised Anxiety Disorder; PHQ-2, Patient Health Questionnaire; TAU, treatment as usual; VIP, Victim Improvement Package.

At baseline, data on MINI diagnosis were available in 82% (107/131) of participants. The most common MINI diagnosis was major depressive disorder which was 68.2% (73/107). 46.6% (48/103) were recorded as current cases, 34.3% (36/105) as past and 24.0% (25/104) as recurrent. Other diagnoses included 48.0% (49/102) of Generalised Anxiety Disorder (GAD), 25.3 (24/95) of post-traumatic stress disorder (PTSD), 46.1% (47/102) of lifetime panic disorder and 36.7% (36/98) of current panic disorder.

Table 3 shows BDI-II and BAI scores and the composite measure for each of the timepoints, and their treatment effects. Data with mean imputation were available in 67.2% (88/131) and 61.1% (80/131) post-intervention and follow-up respectively. Alcohol abuse was low, with only one person in the TAU group at baseline and one person in the VIP group at baseline reporting abusing alcohol.

**Table 3:** Primary and secondary outcomes with mean imputation, when applicable

Variable	Time	VIP (n=65)	TAU (n=66)	Difference or OR adjusting for baseline (6 months) (95% CI)
		Mean (SD) or n/n’ (%)	Mean (SD) or n/n’ (%)	
Beck Depression Inventory-II (BDI-II)	Baseline (3 months)	19.8 (11.4)	23.0 (12.0)	
	6 months follow-up	16.6 (10.7) (n’=40)	19.7 (13.4) (n’=48)	0.099 (−3.752, 3.951)
	9 months follow-up	19.1 (11.7) (n’=39)	17.8 (12.0) (n’=40)	1.946 (−2.291, 6.184)
Beck Anxiety Inventory (BAI)	Baseline (3 months)	22.7 (13.5)	21.7 (12.4)	
	6 months follow-up	17.7 (11.4) (n’=40)	18.0 (11.8) (n’=48)	0.658 (−3.753, 5.069)
	9 months follow-up	18.9 (10.4) (n’=39)	19.2 (13.7) (n’=41)	−0.251 (−5.241, 4.738)
Composite outcome	Baseline (3 months)	−0.07 (0.97)	0.08 (1.01)	
	6 months follow-up	−0.41 (0.89) (n’=40)	−0.19 (1.11) (n’=48)	−0.039 (−0.386, 0.308)
	9 months follow-up	−0.16 (0.98) (n’=39)	−0.29 (1.06) (n’=41)	0.189 (−0.184, 0.561)
MINI caseness*				
Major depressive episode	Baseline (3 months)	36/53 (67.9%)	37/54 (68.5%)	
	6 months follow-up	16/35 (45.7%)	32/44 (72.7%)	0.244 (0.037, 1.588)
Panic disorder—lifetime	Baseline (3 months)	22/51 (43.1%)	24/50 (48.0%)	
	6 months follow-up	7/27 (25.9%)	16/37 (43.2%)	0.285 (0.019, 4.246)
Panic disorder—current	Baseline (3 months)	19/49 (38.8%)	17/48 (35.4%)	
	6 months follow-up	3/31 (9.7%)	6/35 (17.1%)	0.454 (0.086, 2.398)
Post-traumatic Stress Disorder†	Baseline (3 months)	15/47 (31.9%)	9/47 (19.2%)	
	6 months follow-up	6/27 (22.2%)	3/30 (10.0%)	1.662 (0.312, 8.859)
Generalised Anxiety Disorder	Baseline (3 months)	27/51 (52.9%)	21/50 (42.0%)	
	6 months follow-up	11/30 (36.7%)	15/35 (42.9%)	0.792 (0.259, 2.417)

Due to small number of cases, alcohol abuse is not included in the modelling.

* Due to small numbers in some sites, sites are taken out from the mixed models.

† Due to non-convergence, sites are taken out and a fixed (logistic regression model) was used instead.

BDI-II, Beck Depression Inventory-II; MINI, Mini International Neuropsychiatric Interview; TAU, treatment as usual; VIP, Victim Improvement Package.

**Figure 3:**
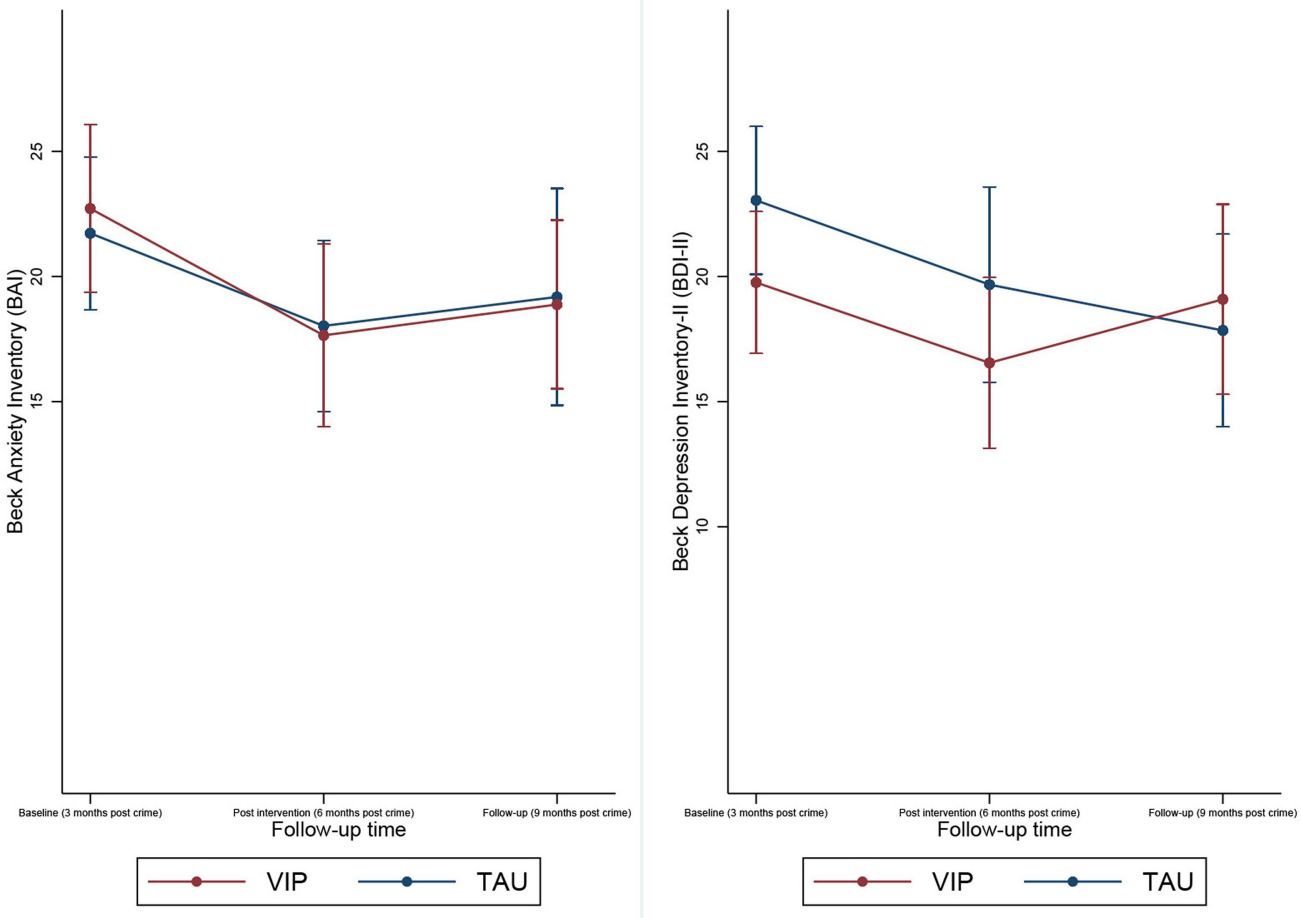
Mean trajectories of BDI-II and BAI respectively over time. BAI, Beck Anxiety Inventory; BDI-II, Beck Depression Inventory-II; TAU, treatment as usual; VIP, Victim Improvement Package.

**Figure 4:**
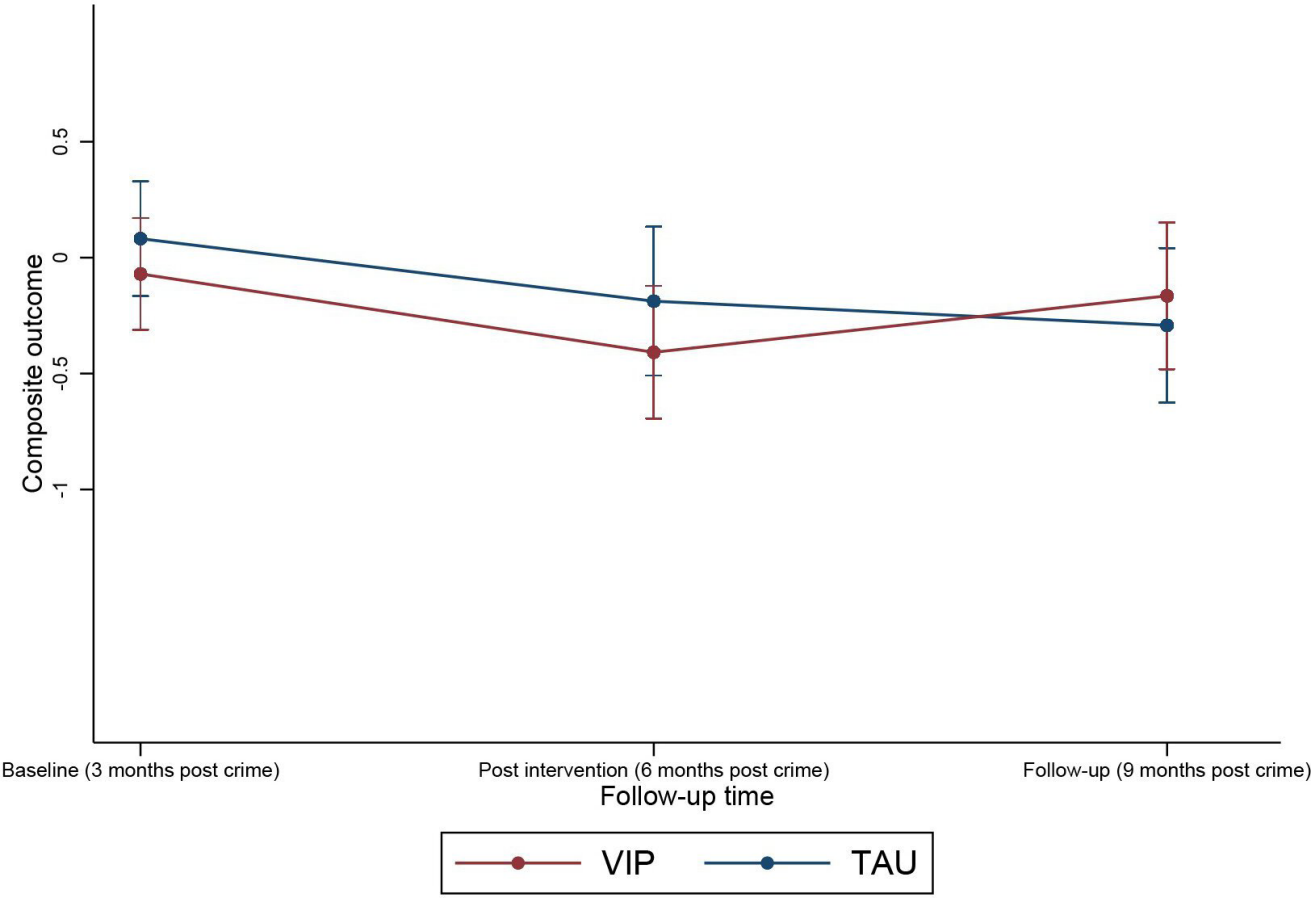
Composite BDI-II and BAI score over time. BAI, Beck Anxiety Inventory; BDI-II, Beck Depression Inventory-II; TAU, treatment as usual; VIP, Victim Improvement Package.

Figure 3 shows the mean trajectories of BDI-II and BAI respectively over time, and figure 4 shows the composite BDI-II and BAI score over time.

### Analysis

We found no treatment effect for the VIP intervention post-intervention (n=88, standardised mean difference −0.039 (95% CI −0.386, 0.308), p=0.821) (table 3) and follow-up (n=80, standardised mean difference 0.189 (95% CI −0.184, 0.561)).

There were no significant effects after stratifying for anxiety or depressive+anxiety symptoms. The ORs for all MINI diagnoses (table 3), except for PTSD, were lower for the VIP arm, although this was non-significant.

### Attrition

67% (88/131) and 61% (80/131) completed data at post-intervention and follow-up respectively, with 74% (97/131) of participants having at least one data point. Eight participants unable to be assessed at post-intervention were seen at follow-up. Six people withdrew from the intervention, because in the TAU group, three participants were unhappy about group allocation and in the VIP group, three were unhappy about the delay in therapy.

### Data imputation

Living arrangements (p=0.062), GAD-2 (p=0.017) and PHQ-2 (p=0.015) were found to be predictors of missingness at the 10% level. Multiple imputation was used for data post-intervention only due to missingness at follow-up. Best and worst case analyses were performed under the assumptions that all missing data were treated as either having improved or been worse respectively. Stratified analyses suggested a treatment effect for the VIP intervention post-intervention (n=27, standardised mean difference −0.612 (95% CI −1.222, –0.002), p=0.049) for participants who experienced burglary. No other significant treatment effect was found.

### Measures of bias and other measures

For each of the 12 local authority areas (LA), participants were equally allocated to each arm, with the exception of two LAs; in one LA, all (n=7) were randomised to TAU and in another, 80% (4 out of 5) were randomised to VIP. The expectations for improvement with treatment were similar and more favoured the VIP intervention. Satisfaction with the VIP was high, 3.9/5 (SD=1.5) (n=43) (table 4).

**Table 4:** Measure of systematic biases

Variable	VIP (n=65)	TAU (n=66)	Total (n=131)
	Mean (SD) or n (%)	Mean (SD) or n (%)	Mean (SD) or n (%)
Treatment expectation			
Improvement from VIP*	6.5 (3.0) (n’=55)	6.5 (2.5) (n’=55)	6.5 (2.7) (n’=110)
Improvement from TAU*	4.2 (2.8) (n’=55)	4.5 (2.5) (n’=59)	4.4 (2.6) (n’=114)
Receiving any CBT before			
Yes	5 (9.3%)	9 (15.0%)	14 (12.3)
No	49 (90.7%)	51 (85.0%)	100 (87.7%)
Total	54 (100%)	60 (100%)	114 (100%)
Treatment preference			
VIP	30 (53.6%)	36 (60.0%)	66 (56.9%)
TAU	5 (8.9%)	1 (1.7%)	6 (5.2%)
No preference	21 (37.5%)	23 (38.3%)	44 (37.9%)
Total	56 (100%)	60 (100%)	116 (100%)
Treatment blindness—6 months follow-up			
I do not know which group the participant is in	10 (31.3%)	31 (73.8%)	41 (55.4%)
I have guessed the participant is in the VIP group	4 (12.5%)	2 (4.8%)	6 (8.1%)
I have guessed the participant is in the control group	3 (9.4%)	7 (16.7%)	10 (13.5%)
I know that the participant is in the VIP group	13 (40.6%)	0 (0.0%)	13 (17.6%)
I know that the participant is in the control group	2 (6.3%)	2 (4.8%)	4 (5.4%)
Total	32 (100%)	42 (100%)	74 (100%)
Treatment blindness—9 months follow-up			
I do not know which group the participant is in	13 (46.4%)	20 (74.1%)	33 (60.0%)
I have guessed the participant is in the VIP group	4 (16.3%)	0 (0.0%)	4 (7.3%)
I have guessed the participant is in the control group	2 (7.1%)	7 (25.9%)	9 (16.4%)
I know that the participant is in the VIP group	9 (32.1%)	0 (0.0%)	9 (16.4%)
I know that the participant is in the control group	0 (0.0%)	0 (0.0%)	0 (0.0%)
Total	28 (100%)	27 (100%)	55 (100%)

* Treatment expectation is based on self-reported scale between 0 (not at all) and 10 (completely).

CBT, cognitive–behavioural therapy; TAU, treatment as usual; VIP, Victim Improvement Package.

### Measures of quality and adherence of therapy

Of participants allocated to the VIP, 35.4% (23/65) did not receive therapy because: the mental health charity was unable to confirm whether therapy was delivered in nine cases (because of difficulties in accessing files during the pandemic), they did not have capacity in five, three participants withdrew, three were unable to leave home for therapy (before remote delivery was possible), two were not contactable and one was in hospital.

#### Number of therapy sessions and audio-recordings

42 participants received therapy. The majority of participants (63.9%, 53/88) had their treatment delivered face-to-face, 33.1% (30/88) had therapy online and 5.6% (n=5) had a combination of online and face-to-face therapy because of adaptations that needed to be made because of the COVID-19 pandemic. The implications of this are discussed in more detail in our lessons learnt paper.^20^

The number of sessions delivered was available for the period during the COVID-19 pandemic only. Due to limited session data, complier average causal effects (CACE) analysis was not appropriate. A mean of 7.5 (SD 3.6) sessions was delivered. Recordings were available for at least one session of therapy for 21 out of 42 participants and a total of 110 recordings were uploaded. Reasons for not uploading recordings included changes of service structure within the mental health charity, the pandemic and participants’ refusal to consent to sessions being recorded. Some therapists reported during supervision that they did not feel comfortable recording sessions, or were unable to record sessions using digital means when therapy was delivered online.

#### Quality of CBT

16 sessions were independently rated. The mean Cognitive Therapy Scale-Revised (CTS-R) score was 15.8 (SD 16.3). Two ratings achieved competence^31^ with CTS-R scores of 38 and 59, which is above a score of 36 required for competence in CBT.

#### Adherence to the manual

Therapists uploaded a total of 109 TCCs.^26^ The main interventions reported were: setting homework in 61% of sessions, going over the history and impact of the crime in 46% of sessions, focussing on the target complaint in 39% of sessions, using cognitive techniques in 36% and guided discovery in 23%. Our random selection of 16 therapy sessions, independently rated, suggested that the therapist went over the history and impact of the crime in 44% (7/16), used cognitive behavioural techniques in 19% (3/16), guided discovery in 6% (1/16) and set homework in 13% (2/16).

### Serious adverse events

Of the 131 participants randomised, six SAEs were recorded. Five were in the VIP arm; two related to hospitalisation for physical reasons; two for hospitalisation for psychiatric reasons (one under Section 2 of the Mental Health Act and one for an increase in suicidal ideation); with one recorded death, reason unknown. In the TAU group, one participant had an increase in suicidal ideation.

## Discussion

This was the largest impact and intervention study in older victims of community crime^4^ which used the police to identify distress in order to recruit them into an RCT. The police could effectively identify distress as part of routine practice using brief screening tools. Over a third of older victims experienced distress, which was significantly higher than rates expected for this population (7% for depression, 4% for anxiety).^2^ At 3 months, symptoms remained present in almost half, confirming previous findings^5^ that crime is an important public health problem.

Psychological therapy using different deliveries, face-to-face or online, was acceptable and feasible. However, despite consistently lower scores on most MINI mental health outcomes and the primary outcome among those receiving the VIP, these changes were only significant in burglary victims for the primary outcome.

### Identifying the impact of crime and the intervention

The police screened a quarter of community crimes in our target areas and found depressive and anxiety symptoms in 35%. The direction of change in the main outcomes was consistent with our feasibility work^5^ (Serfaty 2016). ORs by MINI diagnoses suggested a lower odds of depression and/or anxiety with VIP but not PTSD. Overall, it was not surprising that these findings were non-significant, given the trial was underpowered. Although we are cautious about interpreting positive findings in an underpowered trial, stratified analyses did suggest decreased anxiety and depression in burglary victims with the VIP. This is consistent with our qualitative findings that burglary is particularly upsetting to its victims.^4 5^ Trauma-related symptoms are hard to treat^32^ and more than 12 sessions may be required.^33^ Our observed treatment effect was also below the minimum clinically important difference of 4 points chosen^16^ and this is addressed below.

### Strengths and limitations

Smaller sample size, differential attrition and quality of therapy may all impact the treatment effects. Other than power considerations, the observed treatment effect being less than expected may be due to:

The attrition rate was between 26% and 32% (compared with our 15% predicted attrition). The COVID-19 pandemic and possibly decreased confidence in the police impacted on recruitment and retention of participants. Dissatisfaction with treatment allocation may be mitigated by offering a credible control treatment, such as a Talking Control,^34^ and delays in accessing treatment may be addressed by using therapists from the independent sector. As found in the results of predictors of missingness, the association between living arrangements, anxiety, depression and/or anxiety, and attrition is complex, and these factors may be related to differential attrition of vulnerable and disadvantaged participants.

While the CTS-R scores do not necessarily predict outcome^31^ and therapists may elect to upload their better sessions, potentially biasing evaluations of therapy, this trial demonstrates the importance of evaluating the fidelity of treatment. Therapists self-reported they were proficient in CBT and adhered to the treatment protocol; however, independent ratings suggested otherwise, with only 2 of the 16 sessions assessed achieving competence in CBT and limited CBT procedures were used from the manual. All but two therapists were from a humanistic background and more training and experience in CBT is recommended.

The COVID-19 pandemic demonstrated that online therapies can be delivered and that older people can engage with technology.^35^ People from poorer socioeconomic backgrounds may benefit by saving on transport costs but be excluded through their limited access to internet services.

Given IAPT services were too overloaded to be able to participate in research and voluntary sector services were in flux mid-trial, employing therapists from the independent sector^36^ may have been preferable, so that participants can receive high-quality therapy promptly. Using self-employed therapists also reduces a number of costs, such as payment for cancelled appointments, and employer overheads. Determining whether an intervention works (efficacy) prior to determining how a treatment is best delivered (effectiveness) is also consistent with the Medical Research Council framework for complex interventions.^37^

### Implications of findings and how they should be applied to victim care and research

Standardised tools were a brief and effective way of identifying distress and could be incorporated in routine SNT visits, but more research is required into how to manage older people once distress is identified, possibly through integrated care pathways,^38^ as the way victims experience the police is complex.^39^ When the analyses were stratified by crime type, there appeared to be a treatment effect with the VIP for burglary victims. Further research should possibly focus on this crime type rather than all common crimes in the first instance.

However, our experience suggests recruiting the numbers required for an RCT through police services may be unrealistic in a rapidly changing policing environment. The use of Single Case Experimental Design (SCED)^40^ may be more suitable as participants act as their own control,^41^ reducing the sample size and improving retention as all participants receive an intervention.^40^ Findings may also be more generalisable.^42^

CBT is a clinically effective intervention^43^ and acceptable to older people.^5 8^ Although satisfaction with the VIP was high, its clinical effectiveness remains to be determined further evaluated.

Although five out of six of the SAEs were from the VIP group, these were mostly related to physical problems. Where psychological distress was present, admission to hospital was accounted for by better detection of distress. Figures3  4 suggested an increase in depressive and anxious symptoms once therapy has finished. It is possible that the ending of treatment may result in an increase in distress^44^; and therapists indicated in supervision that participants requested a continuation of therapy. In this case, ethical considerations stipulated that signposting to existing services should occur.

### Conclusions

Community crime in older people is an important public health problem, with sustained symptoms of distress in half those impacted. Although the police can effectively identify distress using standardised measures, signposting to local services is not effective.^23^ Remote delivery of therapy increases access to care and the VIP package may still be an effective treatment for older victims of crime, especially victims of specific crimes. However, ensuring that only CBT-accredited therapists are used is recommended and evaluating the quality and adherence to the CBT model through independent ratings is essential. Using the police for research to screen and recruit participants into an RCT and delivering CBT in a realistic setting are challenging. Alternative trial designs and the use of independent therapists who have the skills and capacity to deliver CBT should be considered in future research.

## Supplementary material

10.1136/bmjopen-2024-095184 online supplemental file 1

## Data Availability

Data may be obtained from a third party and are not publicly available.
