# Supplementary material for: The VIP trial: a randomised controlled trial of the clinical effectiveness of a Victim Improvement Package (VIP) for the reduction of continued symptoms of depression or anxiety in older victims of community crime in an English city
Source: BMJ Open. 2025 Jul 25;15(7):e095184. doi: 10.1136/bmjopen-2024-095184 (PMC12306311; doi:10.1136/bmjopen-2024-095184)
Supplement: Supplementary file 2 [file bmjopen-2024-095184.reviewer_comments.pdf]

PEER REVIEW HISTORY

BMJ Open publishes all reviews undertaken for accepted manuscripts. Reviewers are asked to complete a checklist review form and are provided with free text boxes to elaborate on their assessment. These free text comments are reproduced below.

ARTICLE DETAILS

Title (Provisional)

The VIP trial: a randomised controlled trial of the clinical effectiveness of a Victim Improvement Package (VIP) for the reduction of continued symptoms of depression or anxiety in older victims of community crime in an English city.

Authors

Serfaty, Marc; Satchell, Jessica; Lee, Teresa; Laycock, Gloria; Brewin, Chris; Buszewicz, Marta; Leavey, Gerard; Drennan, Vari M.; Vickerstaff, Victoria; Cooke, Jonathan; Kessel, Anthony

---

VERSION 1 - REVIEW

**Reviewer**	**1**
**Name**	**Dack, Kyle**
**Affiliation**	**University of Bristol, MRC Integrative Epidemiology Unit**
**Date**	**19-Nov-2024**
**COI**	**None**

The authors demonstrate that this topic is understudied and clinically important, and I agree that it is important to share the results despite the premature end to the trial (due to world events).

Unfortunately the tables and figures seem to be missing so I have performed a statistical review as best I could, but it is incomplete. The methodology seems valid from what I can infer, and the paper is otherwise well written.

Abstract

1. I suggest reporting the study location in the setting.

2. GAD-2, PHQ-2 and CBT are referenced before the acronyms are defined. If short of words, the exclusion criteria could be removed or shortened - it is not essential to the STROBE for abstracts checklist.

3. “131 out of our target of 226 people were randomised, 66 in the TAU arm and 65 in the TAU plus VIP. 67% (88/131) of the randomised participants were included in the primary

analysis.” Suggest removing this from the “participants” section, because it is repeated in the results.

4. “68% (89/131) completed the primary outcome (post-intervention)” – the primary outcome is listed as 3, 6, and 9 months – is the 68% those who completed all 3 timings? Similarly when reporting the estimated change, the timing is unclear.

Introduction

5. The study protocol reports that the study is located in 9 London boroughs. However, in this manuscript doesn’t seem to be mentioned, not even the country, e.g. “we collaborated with the police force and a national mental health charity in a large metropolitan area, to develop the VIP trial”. Unless there are reasons related to anonymity I think the paper would benefit from including this information to help readers understand the context of the study.

Objectives

6. Similar to the comment in the abstract, it would be helpful here or somewhere else to clarify the timeframe involved in this evaluation, because this will help the reader to understand the subsequent methods section.

Methods

7. “Police-reported victims of community crime aged 65 and over, with continued symptoms of anxiety and/or depression were identified.” Suggest adding “(using the procedure described below)” or similar.

8. PHQ-2 and GAD-2 should be briefly explained before using the acronyms.

9. It may be explained the missing figure, but it would be helpful to explicitly state somewhere who performed the symptom screening. Steps 1 states that various police staff screened participants but I’m not sure if this means only screening crime type or also the symptoms of distress.

10. Step 2 states that participants were “rescreened” for distress symptoms by a university researcher. Again, I’m not sure if this means symptom screening was also a part of step 1 or not. Maybe adding a high-level summary of the three steps and what was measured at each point would help clarify these points.

11. Step 3 inclusion/exclusion criteria : “Victims of community crime aged 65 years or more” this is part of the initial inclusion criteria so it seems redundant to have it again – suggest reporting only the new criteria at this stage.

12. “The VIP intervention is summarised in 16” Please also add a summary to this paper because it is an integral part of the methodology and required for STROBE item 5.

13. Why were different depression/anxiety scales used for screening compared to outcome measurement? I am not familiar with these scales and a brief explanation would be welcome.

14. “Reported elsewhere, EuroQol 28 and a modified Client Service Receipt Inventory (CSRI)29” It is not clear what this means.

Results

15. The mean difference is reported to three decimal places – is this clinically meaningful or could two decimal places be used?

16. I have not reviewed tables/figures but the in-text results are reported clearly.

Discussion

17. I have seen similar RCTs which were impacted by the Covid-19 pandemic, and the authors used the papers to share what they learned about performing an RCT under such difficult circumstances. I imagine the authors of this RCT may also have such experiences, and it could be a valuable part of the discussion.

**Reviewer**	**2**
**Name**	**Valdes, Olivia**
**Affiliation**	**FINRA Investor Education Foundation, Washington**
**Date**	**20-May-2025**
**COI**	**None**

Thank you for the opportunity to review this paper. The authors describe a randomised controlled trial to examine the effectiveness of a Victim Improvement Package therapy program for victims for community crime in terms of chronic distress. The findings suggest no benefits VIP compared to TAU. However, there is some evidence of effects for burglary-related crime victims. Overall, this study is interesting and has important implications. However, the limitations, in large part acknowledged by the authors create interpretation challenges that are difficult to address.

I was unable to see the supplementary materials or any figure and tables in this attachment. I was only able to see other cited and relevant work which limited my ability to make an assessment on the results and methodology.

Large levels of nonrandom attrition are problematic and it is not clear whether the attrition was differential or how this was addressed. It's possible that (especially amid the COVID-19 pandemic) more vulnerable and disadvantaged participants withdrew from the study and biased the results.

Unclear what the effect of face-to-face therapy vs. online therapy was and particularly the option of deciding what kind of therapy to participate in.

The sample size of participants who experienced burglary was very small and it is unlikely that there is sufficient power to draw conclusions from an n=27.

Variability in the delivery and quality of therapy and therapists. The poor ratings in CBT show that the therapists' experience in CBT may have created some challenges for therapy. Are there any additional therapist-level controls or variables that can be examined?

VERSION 1 - AUTHOR RESPONSE

REVIEWER: 1
Dr. Kyle Dack, University of Bristol
Comments to the Author:

GENERAL COMMENT.
The authors demonstrate that this topic is understudied and clinically important, and I agree that it is important to share the results despite the premature end to the trial (due to world events).

RESPONSE.
Thank you

GENERAL COMMENT.
Unfortunately the tables and figures seem to be missing so I have performed a statistical review as best I could, but it is incomplete. The methodology seems valid from what I can infer, and the paper is otherwise well written.

RESPONSE:
Thank you for feeding back that the paper is well written. The tables and figures were uploaded and pdf did include the tables at the end. We have now inserted the tables within the manuscript and the figures at the end as recommended.

COMMENT 1.
Abstract
1. I suggest reporting the study location in the setting.

RESPONSE 1.
Thank you. We have added the location in the title and the following sentence:

> **Setting:** Police-reported victims of community crime aged 65 and over were recruited from 12 local authority areas.

This has been revised to

> **Setting:** Police-reported victims of community crime aged 65 and over were recruited from 12 local authority areas in a major urban city in England, UK.

COMMENT 2.
2. GAD-2, PHQ-2 and CBT are referenced before the acronyms are defined. If short of words, the exclusion criteria could be removed or shortened - it is not essential to the STROBE for abstracts checklist.

RESPONSE 2:
Thank you for being mindful of the need to keep within the word count. We have expended the acronyms and maintained the meaning.

The following:

>**"Participants:** Selection criteria-Inclusion: Victims of community crime aged 65 years or more, with significant GAD-2 and PHQ-2 distress associated with the crime. Exclusion: Victims of sexual violence or domestic violence; a self-reported diagnosis of schizophrenia or bipolar disorder; a MINI diagnosis of alcohol dependency; receipt of CBT in the last 6 months; an inability to participate in CBT; cognitive impairment."

Has been revised to:

>***"Participants:** Selection criteria-Inclusion: Victims of community crime aged 65 years or more, with significant generalised anxiety disorder (GAD-2) and patient health questionnaire (PHQ-2) distress associated with the crime. Exclusion: type of crime, diagnosis, receipt of Cognitive Behaviour Therapy (CBT) in the last 6 months; an inability to participate in CBT; cognitive impairment".*

COMMENT 3.

3. "131 out of our target of 226 people were randomised, 66 in the TAU arm and 65 in the TAU plus VIP. 67% (88/131) of the randomised participants were included in the primary analysis." Suggest removing this from the "participants" section, because it is repeated in the results.

RESPONSE 3:

We have removed the line above as suggested. However, we wished to be transparent about our trial being underpowered and have changed the line in our results from

>*"131 were randomised";*

Has been revised this to:

>*"Out of our target of 226 participants, only 131 were recruited and randomised".*

COMMENT 4.

4. "68% (89/131) completed the primary outcome (post-intervention)" – the primary outcome is listed as 3, 6, and 9 months – is the 68% those who completed all 3 timings? Similarly when reporting the estimated change, the timing is unclear.

RESPONSE 4:

We are sorry that this was unclear. All timings are with respect to the crime. Baseline was at 3 months, the primary endpoint was post the VIP intervention (6 months) and the followup was at 9 months post crime.

The following:

>***"Primary and secondary outcome measures:** Primary outcomes: a composite of the Beck Anxiety and Beck Depression Inventories, collected at baseline, post intervention and follow-up (3-, 6- and 9-months post crime)."*

Has been revised to:

>***"Primary and secondary outcome measures:** timings are in relation to the crime; baseline (3 months), post VIP intervention (6 months), and followup (9 months). The primary outcome was a composite of the Beck Anxiety and Beck Depression Inventories. The primary endpoint was 6 months."*

COMMENT 5.

Introduction

5. The study protocol reports that the study is located in 9 London boroughs. However, in this manuscript doesn't seem to be mentioned, not even the country, e.g. "we collaborated with the police force and a national mental health charity in a large metropolitan area, to develop the VIP trial". Unless there are reasons related to anonymity I think the paper would benefit from including

this information to help readers understand the context of the study.

RESPONSE 5:
The protocol referred to 9 boroughs. As we wished to remain anonymous, for the reasons below, we refer to local authority areas as the term borough would suggest London. In the actual study we expanded to 12 boroughs (local authority areas). The following:

> *"The study was conducted in a large urban area, including inner and outer city areas involving up to 12 local authorities selected for the range of demographic characteristics of their populations".*

Has been revised to

> *"The study was conducted in a large urban area, including inner and outer city areas expanded to12 local authority areas selected for the range of demographic characteristics of their populations."*

Whilst we agree with the reviewer in part, we considered this issue at length in both TMG and TSC meetings and also had an input from the PPI members.

The unanimous view was that naming the police and the charity would not change the conclusions. Furthermore, public criticism would be likely to deter would be institutions for collaborating in research. The reality is that if readers really wish to work out both the police force and the mental health charity, they could potentially do so, but for the average reader we wished maintain discretion.

Some of the challenges are published in our lessons learned paper (Serfaty et al, 2025) which form part of this threaded publication (https://doi.org/10.23310/KGMR6521).

We have added this reference for the lessons learned paper to for the editor (see response below).

Serfaty, M., Satchell, J., Laycock, G. K., Brewin, C. R., Buszewicz, M., Leavey, G., Drennan, V. M., Cooke, J., & Kessel, A. (2025). Cross-agency working when conducting a pragmatic RCT for older victims of crime: our experiences and lessons learned. *Trials*, *26*(1), 17. https://doi.org/10.1186/s13063-024-08680-y

---

COMMENT 6.
Objectives
6. Similar to the comment in the abstract, it would be helpful here or somewhere else to clarify the timeframe involved in this evaluation, because this will help the reader to understand the subsequent methods section.

RESPONSE 6:
Three screening methods were undertaken at different times and it may be misleading without breaking this down as we did in detail the methods.

> *"Screen methodology 1 (SM1) (June 2017 -August 2019), In methodology 2 (SM2) (May 2021-June 2022), In methodology 3 (SM3), (March 17th March to 30th June 2023). We would be happy to add the timeframe in the background, although on balance it seems repetitious".*

However, under the section eligibility and screening the following:

> *As the trial progressed, three different approaches were used for screening, adapting our methods to changing circumstances.*

Has been revised to:

As the trial progressed, three different screening approaches (intermittently between June 2017-June 2023) were used, adapting our methods to changing circumstances (Serfaty et al, 2025).

COMMENT 7.

Methods

7. "Police-reported victims of community crime aged 65 and over, with continued symptoms of anxiety and/or depression were identified." Suggest adding "(using the procedure described below)" or similar.

RESPONSE 7:

Thank you. The following:

"Police-reported victims of community crime aged 65 and over, with continued symptoms of anxiety and/or depression were identified".

Has been revised to:

"Police-reported victims of community crime aged 65 and over, with continued symptoms of anxiety and/or depression were identified using the procedures described below".

COMMENT 8.

8. PHQ-2 and GAD-2 should be briefly explained before using the acronyms.

RESPONSE 8:

We have spelt out the acronyms in the abstract. In the methods the following sentence

"Victims were defined as screen positive for distress if they had significant depressive (≥ 3 for the PHQ-2) [18] and/or anxiety symptoms (≥2 for the GAD-2) [19]."

Has been revised to:

"We used the Patient Health Questionnaire (PHQ-2) [18] and Generalised Anxiety Disorder (GAD-2) [19] questionnaire for screening. Each question scores from 0 to 3. The total PHQ-2 is the sum of the scores (range 0-6). The GAD-2 also consists of two questions and is scored in the same way. Victims were defined as screen positive for distress if they had significant depressive (≥ 3 for the PHQ-2) and/or anxiety symptoms (≥2 for the GAD-2)".

COMMENT 9.

9. It may be explained the missing figure, but it would be helpful to explicitly state somewhere who performed the symptom screening. Steps 1 states that various police staff screened participants but I'm not sure if this means only screening crime type or also the symptoms of distress.

RESPONSE 9:

Who performed the screening is described under step 1 police screening. For clarity the sentence in screen methodology 3 the following

"In methodology 3 (SM3), (March 17th March to 30th June 2023) two police administrators made telephone contact with the victim and, with their agreement, passed their details to a specialist university researcher integrated within the police service who could provide them with information about the trial and direct SNTs to undertake a home visit using the same procedures as in SM1."

Has been revised to:

"In methodology 3 (SM3), (March 17th March to 30th June 2023) two police administrators made telephone contact with the victim and, with their agreement, passed their details to a specialist university researcher integrated within the police service who screened participants

for distress and then provided them with information about the trial and direct SNTs to undertake a home visit using the same procedures as in SM1."

COMMENT 10.

10. Step 2 states that participants were "rescreened" for distress symptoms by a university researcher. Again, I'm not sure if this means symptom screening was also a part of step 1 or not. Maybe adding a high-level summary of the three steps and what was measured at each point would help clarify these points.

RESPONSE 10.

We are sorry that the figures and tables were missing and this was unclear. Outline is we hope nicely shown in figure 1 and we have clarified this under the point above. Rescreening was part of step 2. The following:

> *"Those screening positive at Step 1 were rescreened for significant distress by a university researcher using the PHQ-2 and GAD-2. This was initially through home visits then by telephone during the COVID-19 pandemic".*

Has been revised to:

> *"Those screening positive at Step 1 were rescreened for significant distress by a university researcher as part of step 2 using the PHQ-2 and GAD-2. This was initially through home visits then by telephone during the COVID-19 pandemic".*

COMMENT 11.

11. Step 3 inclusion/exclusion criteria : "Victims of community crime aged 65 years or more" this is part of the initial inclusion criteria so it seems redundant to have it again – suggest reporting only the new criteria at this stage.

RESPONSE 11:

Thank you for the suggestion. In some trials the inclusion criteria are not always the same as the screening criteria. In this case we could add the same criteria as used in screening, but then the reader would need to scroll up in the text and so we feel the current layout makes it clearer and so have left it as is? If suggested, we would be happy to change the sentence, but on balance think the current format is justified.

> *"Inclusion: Victims of community crime aged 65 years or more, with scores above cut offs on the GAD-2 and PHQ-2 and who associated their distress with the crime".*

Could be revised to:

> *"Inclusion: As outlined in screening section above".*

COMMENT 12.

12. "The VIP intervention is summarised in 16" Please also add a summary to this paper because it is an integral part of the methodology and required for STROBE item 5.

RESPONSE 12:

The folllowing

> *"The VIP intervention is summarised in [16] and the full manual, available from the Chief Investigator [15]."*

Has been revised to:

> *"The VIP intervention consists of Up to 10 manualised individual sessions were delivered over 3 months by mental health charity therapists. These are: Session 1: Crime narrative,*

underlying beliefs, behaviour; Session 2: Psychoeducation about crime and CBT; sessions 3-8: mood diaries, guided discovery, behavioural experiments; sessions 9-10: relapse prevention. These are summarised further in [16] and in the full manual, available from the Chief Investigator [15]."

COMMENT 13.

13. Why were different depression/anxiety scales used for screening compared to outcome measurement? I am not familiar with these scales and a brief explanation would be welcome.

RESPONSE 13:

The police are undertaking the screening and are time limited. The GAD-2 and PHQ-2 take 2-3 minutes to complete, whereas the BDI-II and BAI can take up to 20 minutes or more with older people. With 4,255 people screened, this would take (17 x 4255 = 72,335 minutes) 1,206 extra hours of time. This would not be realistic or feasible. The GAD-2 and PHQ-2 have been demonstrated to be reliable screening tools for anxiety and depression respectively and we have referenced this in the eligibility and screening section. However, these scales are unlikely to be sensitive enough for the purpose of an RCT.

Some victims of crime present with anxiety related symptoms and others present with depressive symptoms (with or without anxiety). It is however, impossible to know whether victims will have predominantly anxiety or depressive symptoms, as this depends on their cognitive set; i.e. how they view the world. The Beck Anxiety Inventory is good at detecting anxiety related symptoms and the Beck Depression Inventory is good at picking up depression related symptoms. These scales also have far more cognitive elements than other more biologic be related skills used to measure these symptoms in drug trials. Some patients may even have a combination of these symptoms. Rather than make assumptions about the predominant presentation, we chose to use a composite measure of these two scales.

COMMENT 14.

14. "Reported elsewhere, EuroQol 28 and a modified Client Service Receipt Inventory (CSRI)29" It is not clear what this means.

RESPONSE 14:

The following:

Reported elsewhere, EuroQol [28] and a modified Client Service Receipt Inventory (CSRI)[29].

Has been revised to

We collected health economics data using the EuroQol [29] and a modified Client Service Receipt Inventory (CSRI)[30]. These findings will be published separately.

Please note the health economics paper is due to be in press.

COMMENT 15:

Results

15. The mean difference is reported to three decimal places – is this clinically meaningful or could two decimal places be used?

RESPONSE 15:

This is statistically meaningful Three decimal places were chosen due to small numbers (e.g. -0.039) to preserve precision.

COMMENT 16:
16. I have not reviewed tables/figures but the in-text results are reported clearly.

RESPONSE 16.
We are sorry the tables/figures did not come up. Thank you for bringing this to our attention. We have included them in the body of the text.

COMMENT 17.
Discussion
17. I have seen similar RCTs which were impacted by the Covid-19 pandemic, and the authors used the papers to share what they learned about performing an RCT under such difficult circumstances. I imagine the authors of this RCT may also have such experiences, and it could be a valuable part of the discussion.

RESPONSE 17:
Thank you. Because of limited space we could not put all this in. We have however published the valuable lessons learned and these are published in the attached paper for the editor and we have added the reference below to the manuscript:

Serfaty, M., Satchell, J., Laycock, G. K., Brewin, C. R., Buszewicz, M., Leavey, G., Drennan, V. M., Cooke, J., & Kessel, A. (2025). Cross-agency working when conducting a pragmatic RCT for older victims of crime: our experiences and lessons learned. *Trials*, *26*(1), 17. https://doi.org/10.1186/s13063-024-08680-y

----

REVIEWER: 2
Dr. Olivia Valdes, FINRA Investor Education Foundation, Washington
Comments to the Author (GENERAL):
Thank you for the opportunity to review this paper. The authors describe a randomised controlled trial to examine the effectiveness of a Victim Improvement Package therapy program for victims for community crime in terms of chronic distress. The findings suggest no benefits VIP compared to TAU. However, there is some evidence of effects for burglary-related crime victims. Overall, this study is interesting and has important implications. However, the limitations, in large part acknowledged by the authors create interpretation challenges that are difficult to address.

RESPONSE (GENERAL)
Thank you. We are indeed aware of the limitations and are pleased the reviewer found the paper interesting.

COMMENT 1.
I was unable to see the supplementary materials or any figure and tables in this attachment. I was only able to see other cited and relevant work which limited my ability to make an assessment on the results and methodology.

RESPONSE 1:

We are sorry that these were not visible. We have inserted them in the text with the resubmission. However, the main paper is uploaded with tables and figures as separate documents. For convenience, we have also included a complete paper with tables and figures and revisions shown in red.

COMMENT 2.
Large levels of non-random attrition are problematic and it is not clear whether the attrition was differential or how this was addressed. It's possible that (especially amid the COVID-19 pandemic) more vulnerable and disadvantaged participants withdrew from the study and biased the results.

RESPONSE 2:
The following sentence was added in the discussion to discuss how missingness are associated with living arrangements, anxiety and depression:

Page 29:
As found in the results of predictors of missingness, the association between living arrangements, anxiety, depression and/or anxiety, and attrition is complex, and these factors may be related to differential attrition of vulnerable and disadvantaged participants.

COMMENT 3.
Unclear what the effect of face-to-face therapy vs. online therapy was and particularly the option of deciding what kind of therapy to participate in.

RESPONSE 3.
This is a really interesting question.

All participants recruited prior to COVID-19 had their therapy delivered face to face. Prior to the pandemic service providers of therapy were reluctant for this to be delivered on line or assumed that older people would not engage. During the COVID-19 pandemic, almost all initially had their therapy delivered on line, with a limited number in the therapy centres with social distancing procedures. Then the pandemic relaxed and hybrid therapy was offered.

We were limited with space and so did not include the information in detail here. Below this question is answer from our lessons learned paper (Serfaty et al 2025). The following:

> *"The majority of participants (63.9%, 53/88) had their treatment delivered face to face, 33.1% (30/88) had therapy on-line and 5.6% (n=5) had a combination of online and face to face therapy".*

Has been revised to:

> *"The majority of participants (63.9%, 53/88) had their treatment delivered face to face, 33.1% (30/88) had therapy on-line and 5.6% (n=5) had a combination of online and face to face therapy because of adaptations that needed to be made because of the COVID-19 pandemic. The implications of this are discussed in more detail in our lessons learned paper (Serfaty et al, 2025) ".*

Serfaty, M., Satchell, J., Laycock, G. K., Brewin, C. R., Buszewicz, M., Leavey, G., Drennan, V. M., Cooke, J., & Kessel, A. (2025). Cross-agency working when conducting a pragmatic RCT for older victims of crime: our experiences and lessons learned. *Trials*, *26*(1), 17. https://doi.org/10.1186/s13063-024-08680-y

Out of interest, the relevant section discussing this in more detail in this paper.

> *Mode of delivery of therapy* The therapy was initially delivered in-person at community centres, but it was necessary to change this to on-line therapy in response to the Covid-19 pandemic. Although there were concerns about older victims' access to computers and ability to use them for remote therapy, the numbers of older people using the internet is growing (43).
> *Lesson learned 3:* The COVID-19 pandemic demonstrated that, providing on-line therapies can be securely delivered, many older people can engage with technology (37). However, social exclusion because of financial reasons remains a concern. Although we funded taxis for victims who were physically house-bound as part of our trial, in a realistic setting, those from poorer socio-economic backgrounds may not be able to afford the transport required, such as taxis, to attend a therapy centre. Furthermore, this group may not always have the means required for one hour of therapy, delivered weekly, over the internet or telephone. On-line therapy also has the disadvantage of allowing people to avoid leaving the house, which we know can occur after a crime (44) On balance, our first priority was to engage participants in therapy and they can then be encouraged to transition to face-to-face therapy if available later in the treatment".

COMMENT 4:
The sample size of participants who experienced burglary was very small and it is unlikely that there is sufficient power to draw conclusions from an n=27.

RESPONSE 4:
We agree and hence we were cautions in our sentence:

> *"Although we are cautious about interpreting positive findings in an underpowered trial, stratified analyses did suggest decreased anxiety and depression in burglary victims with the VIP".*

COMMENT 5.
Variability in the delivery and quality of therapy and therapists. The poor ratings in CBT show that the therapists' experience in CBT may have created some challenges for therapy. Are there any additional therapist-level controls or variables that can be examined?

RESPONSE 5:
Trials rarely measure the fidelity of delivery of therapy. This is a strength of the study in that we measured therapist competence using the Cognitive Therapy Scale-Revised (CTSR) and the Therapy Component Checklist (TCC), which was very revealing. We also collected information about the therapists training and background which is reported in the NIHR report (https://doi.org/10.23310/KGMR6521) (in press).

As highlighted in our lessons learned paper (Serfaty et al, 2025)

> "We aimed to use therapists trained in CBT (BABCP accredited/accreditable), but in practice, although therapists described themselves as being able to deliver CBT, only 3 out of 49 therapists (6%) were formally accredited/accreditable (having undertaken a 2-year course in CBT and practised CBT for 2 years). The remainder were from a humanist background in their core psychotherapy training. Therapists completed a training day, face to face or online, for adapting their CBT skills to older victims of crime and fed-back that this training had increased

their knowledge, skills and confidence with working with older victims. We also encouraged therapists to attend online supervision sessions delivered by MS (the chief investigator), up to twice monthly. As part of a measure of quality control for the research, therapists were also asked to upload recordings of their therapy sessions (Serfaty, Aspden, Satchel et al, 2020) on a secure website to be assessed for quality of using the Cognitive Therapy Rating Scale—Revised (CTS-R) (Blackburn, Garland James et al 2001). Half of the therapy sessions delivered (110/221) were uploaded. A random selection of 15% of recordings (n = 16) of therapy sessions were independently rated using the CTS-R according to the trial protocol (Serfaty, Aspden, Satchel et al, 2020). The mean CTS-R score was 15.8 (s.d. 16.3) which is well below the cut-off for competence of ≥ 36 (Blackburn, Garland James et al 2001). Only two of the 16 ratings achieved competence Branson, Shafran, Myles (2015) with CTS-R scores of 38 and 59".

Our independent and objective assessments provide an important message for healthcare delivery. Governments may contract services to would be providers, but recording and assessing treatment delivery may not be as robust as it should be.

However, as indicated previously we are cautious not to be over critical of collaborators as this is likely to discourage third parties from engaging in research. We do not have additional therapist level controls or variables that can be examined.

References:

Blackburn I-M, Garland A, James IA, Milne DL, Baker C, Reichelt FK, Standart S. THE REVISED COGNITIVE THERAPY SCALE (CTS-R): PSYCHOMETRIC PROPERTIES. Behavioural and Cognitive Psychotherapy. 2001;29(4):431-46.

Branson A, Shafran R, Myles P. Investigating the relationship between competence and patient outcome with CBT. Behaviour research and therapy. 2015;68:19-26.

Serfaty M, Shafran R, Vickerstaff V, Aspden T. A pragmatic approach to measuring adherence in treatment delivery in psychotherapy. Cognitive Behaviour Therapy. 2020;49(5):347-60.

Serfaty M, Aspden T, Satchell J, Kessel A, Laycock G, Brewin CR, et al. The clinical and cost-effectiveness of a Victim Improvement Package (VIP) for the reduction of chronic symptoms of depression or anxiety in older victims of common crime (the VIP trial): study protocol for a randomised controlled trial. Trials. 2020;21(1).

Serfaty, M., Satchell, J., Laycock, G. K., Brewin, C. R., Buszewicz, M., Leavey, G., Drennan, V. M., Cooke, J., & Kessel, A. (2025). Cross-agency working when conducting a pragmatic RCT for older victims of crime: our experiences and lessons learned. *Trials*, *26*(1), 17. https://doi.org/10.1186/s13063-024-08680-y
